# The Estimation of Hydrochloric Acid in Gastric Disorders

**Published:** 1891-07-18

**Authors:** 


### THE PRACTITIONER'S MIRROR AND RETROSPECT.

[*The Editor will be glad to receive offers of co-operation and contributions from members of the profession. All letters should be addressed to* THE EDITOR, THE LODGE, PORCHESTER SQUARE, LONDON, W.]

### MEDICINE.

### THE ESTIMATION OF HYDROCHLORIC ACID IN GASTRIC DISORDERS.

We recently reviewed the subject of acid dyspepsia, and discussed the value and importance to the animal economy of free hydrochloric acid in the stomach, more especially as a necessary element in normal digestion. But the absence or relative deficiency of free hydrochloric acid has been utilised as a useful factor in the more or less obscure diagnosis of cancerous affections of the stomach.

The acidity of the gastric juice is due to the presence of two acids, lactic acid and hydrochloric acid. The lactic acid is always the result of digestive processes, while the hydrochloric acid is the acid of the gastric juice. The hydrochloric acid is probably formed from the chlorides in the blood, but how the decomposition takes place is still an unravelled mystery. The mucous membrane of the stomach, even that of the cardiac end, which is richest in acid secreting cells, is itself alkaline; the hydrochloric acid, then, is formed in the act of secretion.

Without attempting to go into details, or to recall the many methods and tests all having the same end in view, we may state briefly that in applying the tests we give the patient a test meal and withdraw a portion of the contents of the stomach after allowing a definite interval to elapse. Of the many test meals we will take, for instance, that of Ewald, which consists of thirty-five grammes of white bread, a tumbler and a-half of water or weak tea with no milk or sugar. This is given early in the morning on an empty stomach. An hour later a small quantity of the gastric contents are withdrawn. This may be done by means of a Colin's pump, or, better still, by passing a stomach tube, and turning the patient on his right side with his head low. Firm pressure over the left hypochondrium will ensure the evacuation of some of the contents of the stomach, if a portion has not already escaped. The process is not difficult of accomplishment, nor is it so disagreeable to the patient as it sounds. The gastric juice having been withdrawn, must be filtered, and the filtrate tested. Of the many tests for the estimation of free hydrochloric acid, we prefer either Leube's tropæoline test or the Gunzburg phloro-glucine test. In employing the former a watch-glass containing a small quantity of a one per cent. aqueous solution of tropæoline is placed on a white surface, and into this yellow fluid a few drops of the filtered gastric juice are dropped. If free hydrochloric acid is present, a bright carmine-red is produced. The most sensitive test is Gunzburg's phloro-glucine-vaniline test, which is so delicate that it enables one to detect the presence of one-twenty-thousandth part of free acid. A few drops of the salmon-coloured test solution is added to ten to fifteen drops of the filtered gastric juice placed in a capsule. The whole is then gently warmed, and a bright red colouration at the edge of the fluid indicates the presence of hydrochloric acid. Certain precautions, such as the previous elimination of albuminoids and peptones from the filtrate, have to be observed. For estimating the acidity of the gastric juice, Professor Dujardin-Beaumetz gives the following directions: Take ten cubic centimetres of gastric juice, and add a few drops of a solution of phenol-phtaleine, which has the property of changing to a bright red in the presence of a free alkali. Then add, drop by drop, a standard solution of soda—one-tenth—and make your calculation on the basis that one cubic centimetre of the solution neutralises ·003646 of hydrochloric acid. In the normal state it takes from four to six cubic centimetres of this decimal solution of soda to produce the reaction. And for the purpose of testing the digestive power of the gastric juice, you practise with it artificial digestion. For this purpose you will place in a test tube five cubic centimetres of gastric juice and a little block of albumen—from five to six millimetres square. Place the whole in a stove, and expose it to a temperature of about 38 deg. to 48 deg. C. You may make at the same time, for purposes of comparison, artificial digestions by the aid of pepsin.

We have thus briefly indicated some of the methods of testing for the abnormal absence of hydrochloric acid in the stomach, or, if diminished, for estimating its relative deficiency. It was Van den Welden who, ten years ago, first drew attention to the disappearance of free hydrochloric acid from the gastric juice of the stomach, and the first trials made by Riegel confirmed the remarkable value of this sign, for he never once discovered the presence of free hydrochloric acid in the cases of cancer of the stomach examined by him. But in course of time other observations were published, more especially those of Ewald, of Cahn and of Méring, who showed that cancer might exist with conservation of free hydrochloric acid.*

It is indeed inconceivable that as soon as a small nodule of cancer appears in the coats of the stomach or begins to involve the mucous membrane, that the physiological secretion of hydrochloric acid should at once be suddenly stopped. Some observations on thirteen resected cases of cancer of the stomach in Billroth's clinic showed that in only seven was there a diminution or absence of free hydrochloric acid. Moreover, it is an established fact that hydrochloric acid may be absent in many other wasting diseases. When we remember, too, that hydrochloric acid may be absent from the fasting healthy stomach, and varies in amount when present according to circumstances, it is not difficult to imagine the very small reliance to be placed on the test, even if it be correctly applied. There is an old and ready diagnostic sign of equal value, and one which does not require so many tedious precautions nor introduce so many sources of error, viz., the presence of sarcinæ in the contents of the stomach. The presence of sarcinæ probably arises from the absence of the physiological disinfectant, free hydrochloric acid, and probably occurs whenever the acid is absent or greatly diminished in percentage. Sarcinæ, of course, are found in the vomited matters in a considerable number of diseases, but the same objection may be made to the more recent acid test. The fact is in neither test have we a pathognomonic sign of cancer. Either is useful as tending to confirm a diagnosis of cancer made from other and more reliable signs, for both the old and the newer tests fail just where their aid is most wanted, viz., in the early stages. And if about equally reliable, there is no doubt that both the patient and busy practitioner would much prefer the simpler test. What says Professor Dujardin-Beaumetz in reference to these tests for detecting the presence of free hydrochloric acid in the stomach, and other allied tests: "With all these methods, can you do without the clinical examination, and establish your diagnosis and your therapeutical treatment on these data alone? No, gentlemen; and when you come to reali e all the time and pains and care which these chemical examinations require, and the small results which they bring, you will be tempted to exclaim that the mountain has laboured and brought forth a mouse."

* Dujardin-Beaumetz. Bull. Gén. de Ther. Sept. 15, 1890.

